# Effects of Soy–Whey Protein Nutritional Supplementation on Hematopoiesis and Immune Reconstitution in an Allogeneic Transplanted Mice

**DOI:** 10.3390/nu14153014

**Published:** 2022-07-22

**Authors:** Xiaoliang Wu, Qinghua Hou, Zhenyu Zhao, Jing Wang, Yanzhi Guo, Lingang Lu, Juan Han

**Affiliations:** 1 Institute of Food and Nutrition Development, Ministry of Agriculture and Rural Affairs, Beijing 100081, China; wuxiaoliang2022@163.com (X.W.); houqinghua08@163.com (Q.H.); zzyadeline@foxmail.com (Z.Z.); 82101201182@caas.cn (J.W.); guoyanzhi@caas.cn (Y.G.); 2 Institute of Feed Research, Chinese Academy of Agricultural Sciences, Beijing 100081, China; 3 Institute of Vegetables and Flowers, Chinese Academy of Agricultural Sciences, Beijing 100081, China

**Keywords:** protein nutritional support, hematological malignancies, bone marrow transplantation, malnutrition, hematopoiesis, immune reconstitution, soy–whey blended protein

## Abstract

Profound malnutrition and immunodeficiency are serious negative effects of radiotherapy and bone marrow transplantation for hematologic malignancy patients. This study aimed to evaluate the effects of nutritional supplementation with a soy–whey protein mixture on hematopoietic and immune reconstitution in an allogeneic transplant mouse model. Male BALB/c (H-2Kd) mice, 6–8 weeks-old, were divided randomly into five groups and then provided with different protein nutrition support. After 28 days, blood samples, bone marrow, spleen, and thymus were harvested to measure the effects. The results showed that soy–whey blended protein supplements promoted hematopoietic stem cell engraftment, body weight recovery, and the recovery of white blood cells, lymphocytes, and neutrophils; triggered the expansion of hematopoietic stem cells and progenitor cell pools by increasing the numbers of the c-kit^+^ progenitor, Lin-Sca1^+^c-kit^+^, short-term hematopoietic stem cells, and multipotent progenitors; enhanced thymus re-establishment and splenic subset recovery in both organ index and absolute number; improved overall nutritional status by increasing total serum protein, albumin, and globulin; protected the liver from radiation-induced injury, and increased antioxidant capacity as indicated by lower concentrations of alanine aminotransferase, aspartate aminotransferase, malondialdehyde, and 4-hydroxynonenal. This study indicated that soy–whey blended protein as important nutrients, from both plant and animal sources, had a greater positive effect on patients with hematological malignancies to accelerate hematopoiesis and immune reconstitution after bone marrow transplantation.

## 1. Introduction

Bone marrow transplantation (BMT) is an effective stem cell therapy for patients with a variety of hematological malignancies [1]. However, patients who undergo BMT often experience profound immunological complications and high-risk morbidity and mortality due to opportunistic infections [2,3]. Although natural killer cell populations and activity are restored to normal levels within 30 days after BMT [4], months or even years are required to regain complete immune constitution, including T and B cell counts [5,6]. Porrata [7], Markovic and Chang et al. [8] correlated the recovery of immunity, especially the low lymphocyte compartments, with poor overall survival outcomes of BMT patients. Thus, a priority is to develop effective and safe therapeutic strategies to enhance hematopoiesis and immune reconstitution in BMT patients, to prevent opportunistic infections and disease relapse, and to improve overall survival.

Strategies have been proposed to promote reconstitution by the administration of immune modulators, such as interleukin-7 [9], interleukin-21 [10], Ghrelin [11], endothelial progenitor cells [12], and luteinizing hormone-releasing hormone [13]. Although all these therapeutic strategies achieved satisfactory results, the effect of nutritional status on reconstitution has not been studied adequately. BMT patients commonly experience nutritional deficiency, especially of vitamin D, and malabsorption related side-effects [14,15]. Patients who undergo myeloablative therapy prior to BMT usually experience pronounced gastrointestinal disorders, such as anorexia, nausea, diarrhea, and stomatitis, which impair the patient’s ability to receive adequate oral nutrition [16]. The administration of high-dose corticosteroids to manage graft-versus-host disease and antiviral drugs may also lead to malnutrition and malabsorption [17,18]. In addition, Keller et al. observed a negative nitrogen balance caused by increased protein degradation after myeloablative therapy and BMT [19]. These adverse events are likely to worsen clinical outcomes of BMT patients [20].

Studies have shown that adequate and balanced nutrition is pivotal for successful hematopoiesis and immune reconstitution and in counter-balancing catabolic stress following BMT [20,21,22,23]. In addition, protein-rich nutrition may contribute positively to the rapid recovery of the overall physiology of BMT patients. For instance, protein supplementation can be beneficial to the efficient repair of chemo- and radiation therapy-induced tissue damage, to facilitate hematopoiesis, such as the production of white blood cells, red blood cells, and platelets, and to satisfy the needs of hypermetabolism [22].

Whey and soy proteins have a high nutritional value because they possess abundant amounts of essential amino acids [24,25], and they have different rates of digestion. Whey protein is a “fast” protein because it is rapidly hydrolyzed for quick release into plasma [26]. In contrast, soy protein undergoes slow digestion and requires more time to increase the plasma concentrations of free amino acids [27,28]. Hence, the combination of these two proteins to compensate for the shortcomings of the amino acid release rate is likely conducive to the continued supply of amino acids. In addition, Aoki et al. and Tosukhowong et al. showed that the antioxidant properties of soy and whey protein extracts can neutralize oxidative stress-induced free radicals [29,30]. Moreover, these two protein types broaden the range of overall immune defense in humans [31,32,33]. Therefore, we hypothesized that a combination of soy and whey proteins could offer robust immune healing effects compared with effects of individual proteins.

Ren et al. found that soy–whey blended protein ingested before allo-BMT improved the protein-energy malnutrition status, significantly delayed muscle atrophy, and produced a low trend of infection for leukemia patients who received BMT [34]. These findings raise the questions of whether soy–whey blended protein can facilitate hematopoiesis and immune reconstitution after BMT and whether the protein blend is more beneficial than a single protein. To address these questions, we established a mouse model of BMT to assess the effects of protein supplementation. We conducted a four-week protein intervention after irradiation and BMT, and examined the peripheral blood cell counts, immunological parameters, and serum biochemical indicators at different time points (a gradient time course) after BMT. To the best of our knowledge, this study is the first to evaluate the effects of soy–whey blended protein nutritional supplementation on the rate of the recovery of hematopoiesis and immune system reconstitution following BMT.

## 2. Materials and Methods

### 2.1. Animals and Grouping

Male C57BL/6 (H-2Kb) and BALB/c (H-2Kd) mice aged 6 to 8 weeks, weighing 21–23 g, were purchased from Beijing Vital River Laboratory Animal Technology Co., Ltd. (Beijing, China). Mice were housed in a pathogen-free environment (12 h light/dark cycle, temperature 22 ± 2 °C and humidity 40–70%), with five mice per cage. All animals were acclimated for 10 days and fed a standard diet and sterilized water ad libitum. One week prior to the experiment, mice were provided sterilized water containing gentamicin (320 mg/L) until the end of the experiment. The Institutional Animal Care and Use Committee of Institute of Food and Nutritional Development approved this study (the study ethical approval number is IFND-AP-19-10).

BALB/c mice were randomly divided into the following 5 groups: (1) control group without any treatment (Con, *n* = 30); (2) total body irradiation (TBI) and bone marrow transplantation, then given whey protein (WP, *n* = 30); (3) total body irradiation and bone marrow transplantation, then given soy–whey blended double protein (DP, *n* = 30); (4) total body irradiation and bone marrow transplantation followed by administration of sterile saline (NP, *n* = 30); (5) total body irradiation without any other treatment as a test control of effectiveness of TBI and BMT (*n* = 30).

### 2.2. Bone Marrow Transplantation (BMT)

A mouse model of BMT was established as described [11] and modified as required. Briefly, BALB/c mice received lethal total-body irradiation with ^137^Cs gamma-ray of 8.0 Gy at an approximate rate of 0.89 Gy/min (irradiated twice, each with 4Gy at 4 h interval) on day 0. Donor bone marrow mononuclear cells were isolated from tibias and femurs of C57BL/6 mice, and the cell concentration was adjusted to 2.5 × 10^7^/mL. After 4–6 h of irradiation, 5 × 10^6^ bone marrow mononuclear cells were injected via the tail vein into the BALB/c mice.

### 2.3. Protein Supplementation

The volume of DP nutrition intervention was determined based on the maximum solubility of DP (soy:whey = 1:1 (*w*/*w*)) and was composed of soy (YP928H, De Zhou, Shangdong, China) and whey (Hilmar9410, Livingston, CA, USA) protein isolates in saline solution [35,36]. At the same time, we also considered a 1.5 g/kg body weight /d standard protein supplement dose. Because the gastric perfusion volume limit is 0.1–0.2 mL/10 g body weight, the maximum gavage volume was determined as 0.3 mL to adapt to fluctuations in mouse body weight after BMT. First, we measured the solubility of DP in 0.3 mL saline. Then, we confirmed the optimal amount of DP in physiological saline as 34 mg (1:1 (*w*/*w*)). Next, we measured the nitrogen content in whey and soy protein isolates and used a Kieldahl Azotometer (KDY-9820; Scipio, Beijing, China) to balance the nitrogen content between different intervention groups. The results showed that 33.3 mg of WP was equivalent to 34 mg of DP. Therefore, mice in DP and WP groups were administered 34 mg and 33.3 mg of DP and WP, respectively, which was dissolved in 0.3 mL saline. The protein was administered by oral gavage for four weeks starting from the first day after BMT. The NP group of mice was provided an equal volume of saline following BMT.

### 2.4. Peripheral Blood Cell Count

For all experiments, 50 µL of peripheral blood was collected from the orbital sinus on days 7, 14, 21, and 28 after BMT. An automatic hematological analyzer (Mindray, BC-2600, Shenzhen, Guangdong, China) was used to measure blood cell counts.

### 2.5. Organ Index and Cell Count

The spleen and thymus were removed by means of dissection after the mice were euthanized. The residual blood and other fluids on the tissue surface were removed with filter paper, and the wet weights were recorded to calculate the spleen index and thymus index. A gentleMACS Octo Dissociator (Miltenyi Biotec, Cologne, Germany), mouse splenic dissociation kit (Miltenyi Biotec, Cologne, Germany), and gentleMACS 25C Tubes (Miltenyi Biotec, Cologne, Germany) were used to prepare single-cell suspensions, which were passed through a MACS SmartStrainer (Miltenyi Biotec, Cologne, Germany). The cells were collected by centrifugation. Splenocytes were obtained by lysing red blood cells, followed by washing and resuspending. The number of thymocytes, splenocytes, and bone marrow cells was measured with an automatic cell counter (Countstar, BioTech IC1000, Shanghai, China).

### 2.6. Flow Cytometric Analysis

A DxFLEX flow cytometer (Beckman Coulter, Indianapolis, IN, USA) was used to measure lymphocyte subpopulations in peripheral blood and spleen and donor chimerism and hematopoietic stem cells in bone marrow. Anti-mouse CD45(APC-Cy7), CD3(FITC), CD4(PE-Cy7), CD8(Percp), B220^+^(APC), and NK1.1^+^(PE) were used to stain lymphocytes in peripheral blood. Anti-mouse H-2Kd(FITC) and H-2Kb(PE) were used to define donor chimerism. Hematopoietic stem cells and progenitor cells were characterized using an anti-mouse ckit(PE), Sca1(BV510), CD34(PE-Cy7), Flk3(APC) and FITC-conjugated lineage cocktail (CD3e, Gr1, CD11b, B220, TER-119). Lymphocyte subsets in spleen were defined using CD45(APC-Cy7), CD3(FITC), B220^+^(APC). Dead cells were excluded by 7-AAD staining. All antibodies were purchased from BioLegend (San Diego, CA, USA). CytExpert software (Beckman Coulter, Indianapolis, IN, USA) was used to analyze data. The absolute numbers of different lymphocyte subpopulations in peripheral blood and spleen were calculated either according to absolute lymphocyte count or by multiplying splenocytes by the percent of positive cells.

### 2.7. Biochemical Parameters

Required blood samples were collected from mouse tail veins, and the serum was separated by centrifugation. Commercial reagent kits (Shanghai KeHua Bio-Engineering Co., Ltd., Shanghai, China) were used to measure the content of albumin, total protein, alanine aminotransferase, and aspartate aminotransferase in serum on an automatic biochemical analyzer (ZY-1280). The content of malondialdehyde in serum was measured with a commercial reagent kit (Nanjing Jiancheng Bioengineering Institute, Nanjing, China). An ELISA kit (Jinhai Keyu Biotechnology Co., Ltd., Beijing, China) was used to measure 4-Hydroxynonenal. All experimental procedures were performed according to the manufacturers’ instructions.

### 2.8. Quantitative Real-Time Polymerase Chain Reaction

DNA was extracted from whole blood using a genomic DNA extraction kit (TIANMO, Beijing, China) according to the manufacturer’s instructions. A relative quantitative method was adopted to evaluate the expression level of T cell receptor excision circles (TRECs) on day 28 after BMT. Forward (5′-CCAAGCTGACGGCAGGTTT-3′) and reverse (5′-GCATGGCAAGCAGCACC-3′) primer sequences for TRECs amplification were obtained from Ryan et al. [37]. The forward and reverse primers for the control housekeeping gene RAG2 were 5′-TGACGTGGTGTATAGTCGA-3′ and 5′-TCCTGAAGTTCTGGGAGA-3′. The thermal cycling profile for the qRT-PCR reaction (20 µL reaction) was as follows: initial denaturation at 95 °C for 2 min, followed by 40 cycles of amplification (10 s at 95 °C, 30 s at 58 °C, and 30 s at 72 °C) using a QuantStudio^TM^ 6 Flex Real-Time PCR System (Thermo Fisher Scientific, Waltham, MA, USA). All samples were analyzed in triplicate. The relative expression levels of TRECs were expressed as 2^−ΔΔCt^.

### 2.9. Statistical Analysis

All statistical analyses were conducted with SPSS 22.0 software (IBM SPSS, Version 22.0, Armonk, NY, USA), and the data were expressed as the mean ± SEM. The graphics were created using GraphPad Prism 7.0 (GraphPad Software, Inc., San Diego, CA, USA). Data normality tests and homogeneity of variance tests were analyzed first. One-way ANOVA followed by Fisher’s least significant difference multiple range tests were performed to analyze the differences between groups at different time points. *p* < 0.05 was considered statistically significant.

## 3. Results

### 3.1. The Established Allogeneic Transplant Mouse Model, Recipients Successfully Reached Full Bone Marrow Chimerism at 28 Days after BMT

**Figure 1:**
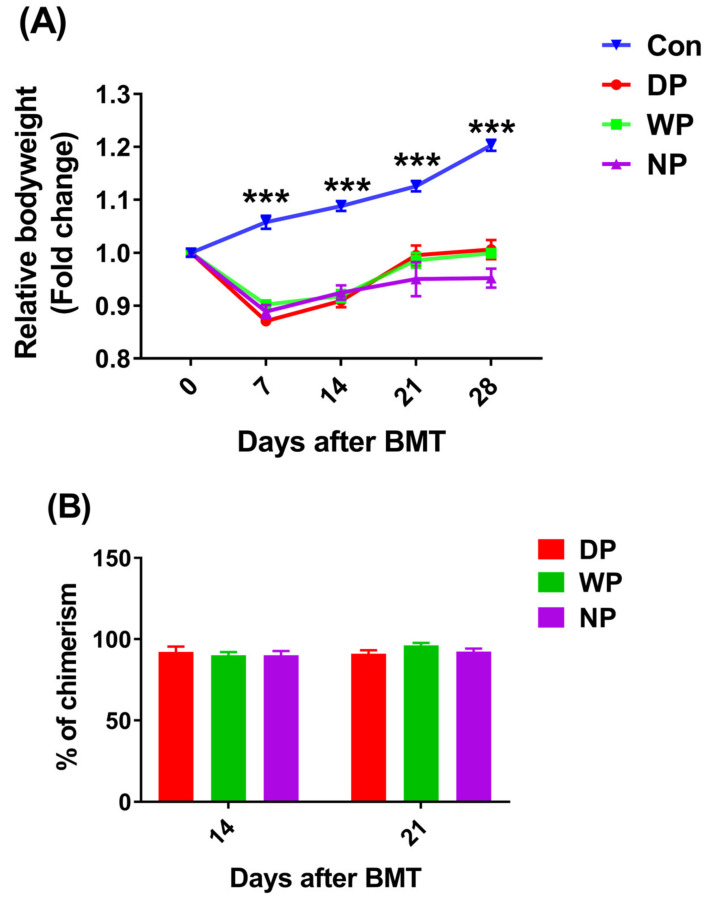
Relative body weight and donor chimerism as a function of time after BMT. BALB/c mice post-BMT were supplemented with DP, WP, or NP. The weights of the mice were obtained at different time points, and relative body weight was calculated (*n* = 23–30) (**A**). Donor chimerism was measured using anti-mouse H-2Kd (FITC) and H-2Kb (PE) on days 14 and 21 (*n* = 6) (**B**). Data are presented as mean ± SEM. Results were obtained from at least two independent experiments. *** *p* < 0.001.

All mice in the TBI group died within 14 days of physical irradiation without BMT therapy; the survival rates of mice in the transplantation groups were not significantly different between groups (data not shown). There were no significant differences in terms of body weight recovery with or without protein supplement post-BMT. However, both DP and WP-treated mice exhibited relatively faster weight recovery compared with recovery of NP-treated mice (*p* > 0.05, Figure 1A). Following transplantation, recipients in DP, WP and NP group reached full bone marrow chimerism at 28 days. On day 14, there part of the BALB/c mice recipients in each BMT treatment group had not yet reached the complete implantation standard of lower than 90%. However, after 21 days, recipients in every group reached full bone marrow chimerism which exceeded 90%, indicating a successful allogeneic bone marrow transplant (Figure 1B and Figure S1).

### 3.2. Hematopoiesis and Lymphocyte Subsets Recovery in Peripheral Blood

**Figure 2:**
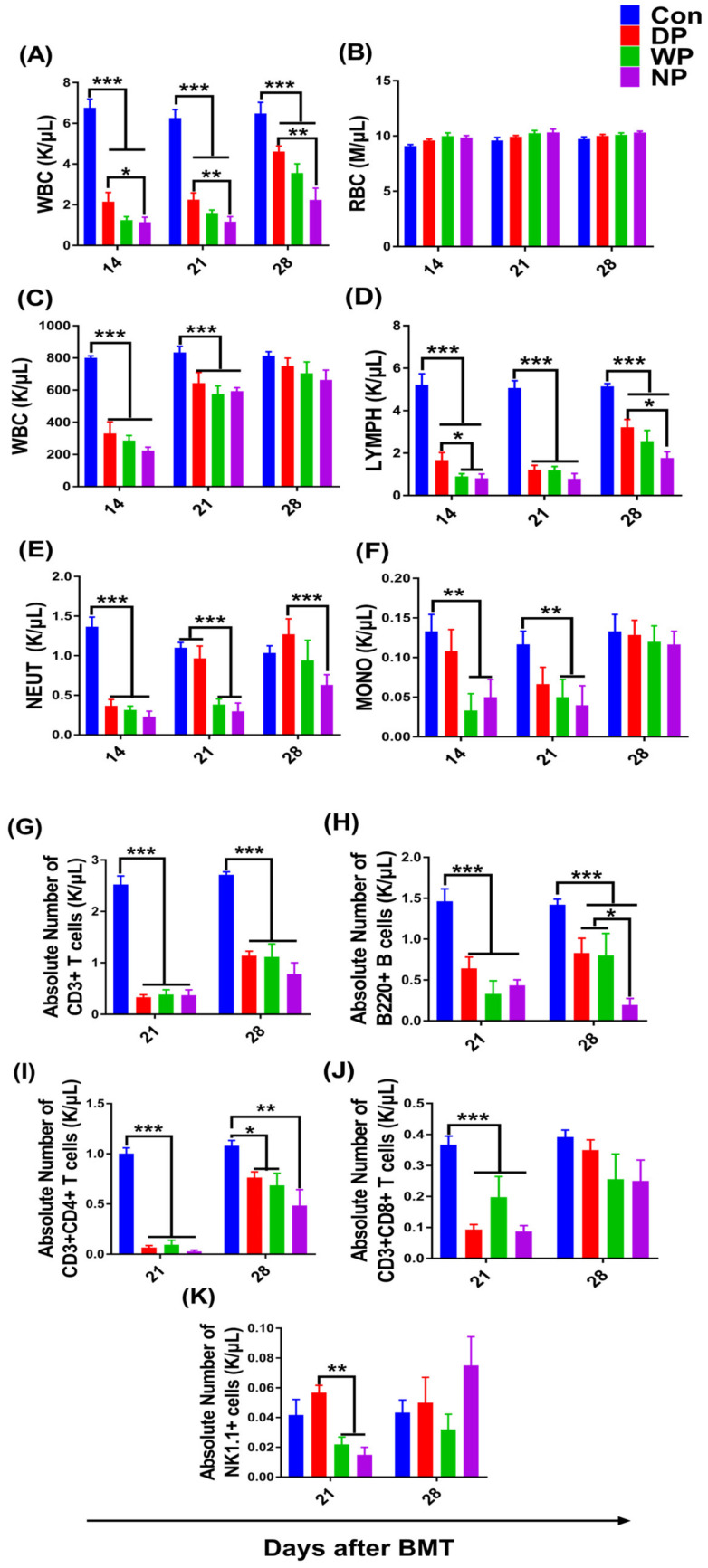
Peripheral blood cell count and lymphoid subsets analysis versus time. Peripheral blood was collected from orbital sinus on day 14, 21, and 28 after BMT for the measurement of cell count including WBC, RBC, PLT, LYMPH, NEUT, and MONO (**A**–**F**); the absolute numbers of CD3^+^, B220^+^, CD3^+^CD4^+^, CD3^+^CD8^+^, and NK1.1 were calculated using flow cytometry results combined with the lymphocyte count in peripheral blood on day 21 and 28 (**G**–**K**). Data are presented as mean ± SEM (*n* = 6). Results are representative of at least two independent experiments. ***p< 0.001, ** *p* < 0.01, * *p* < 0.05.

To examine the effects of different protein supplements on hematopoiesis and immune reconstitution, we measured the peripheral blood cell count on days 7, 14, 21, and 28 after BMT. The number of various types of blood cells, except for red blood cells, dropped to the lowest count on day 7 (data not shown) after TBI and transplantation and then progressively recovered (Figure 2A–F). During post-BMT hematopoietic re-establishment, we found that the white blood cell counts in the DP group were significantly higher compared with the white cell counts in the NP group on days 14, 21, and 28 (*p* < 0.05) (Figure 2A). A high white blood cell count without a large difference in the WP group relative to NP group was observed on days 21 and 28 after transplantation (*p* > 0.05). The higher white blood cell count in the DP group could be explained by increased counts of lymphocytes and neutrophils. Notably, DP supplementation significantly increased the lymphoid recovery in contrast to WP- and NP (saline)-treated mice on days 14 and 28 following BMT (*p* < 0.05; Figure 2D), whereas higher tendencies in lymphoid recovery in WP mice were observed on day 21 and 28 compared with the NP mice. In addition, we measured a higher neutrophil count for the DP group on day 21 and 28 post-BMT compared with the NP group (*p* < 0.01, *p* < 0.05; Figure 2E), and on day 21 compared with the WP group (*p* < 0.01).

Like the reconstitution of blood cell components, various lymphocyte subset counts gradually recovered between day 21 and 28 (Figure 2G–K and Figure S2). Interestingly, on day 28, all the mice in the DP and WP groups had more T cells including CD3+, CD3+CD4+, CD3+CD8+ than mice in the NP group (*p* > 0.05; Figure 2G,I,J). In particular, the numbers of B220^+^ cells in the DP and WP groups were approximately 4-fold higher compared with the NP group (*p* < 0.05; Figure 2H). These results indicated that protein nutrition intervention, especially with soy–whey protein, promoted the recovery of lymphocyte subsets following BMT.

### 3.3. The Recovery of Hematopoietic Stem Cells and Progenitor Cell Pools

**Figure 3:**
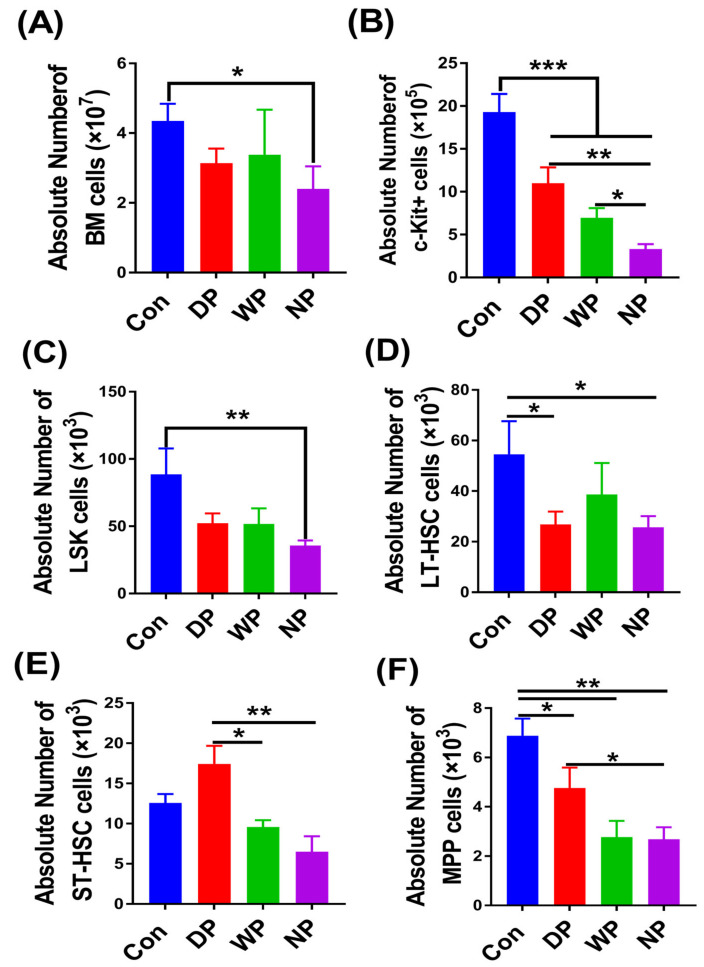
The absolute number of bone marrow cells and hematopoietic stem/progenitor cells (HS/PCs) on day 28 after BMT. Mice were euthanized, and bone marrow cells were harvested for the cell count assay (**A**); simultaneously, the populations of c-kit cells (Lin-c-kit^+^), LSK, LT-HSCs (Lin-Sca1^+^c-kit^+^CD34^−^Flk3^−^), ST-HSCs (Lin-Sca1^+^c-kit^+^CD34^+^Flk3^−^), MPPs (Lin-c-kit^+^Sca1^+^CD34^+^Flk3^+^) were analyzed with well-defined cell surface markers (**B**–**F**). Data are presented as mean ± SEM (*n* = 5–7). Results are representative of at least two independent experiments. *** *p* < 0.001, ** *p* < 0.01, * *p* < 0.05.

To determine whether the peripheral blood cell and lymphoid subset recovery post-BMT as detected in BALB/c recipient mice was a direct effect on bone marrow cells mediated by protein supplementation, we examined hematopoietic stem/progenitor cells on day 28 following BMT using well-defined cell surface markers combined with counting the absolute number of bone marrow cells (Figure S3). Although there was no significant difference in the total bone marrow cell number between the groups (*p* > 0.05; Figure 3A), the number of Lin-c-kit^+^ progenitor cells in DP- and WP-supplemented mice was significantly higher compared to NP treated mice (*p* < 0.01, *p* < 0.05; Figure 3B). The Lin-c-kit^+^ progenitor cell count in DP-supplemented mice was not only highest among the intervention groups but it was also 3-fold greater than in the NP group (*p* < 0.01). The Lin-Sca1+c-kit+ (LSK) counts in DP- and WP-supplemented mice were higher than NP-treated mice, but there was no significant difference (*p* > 0.05; Figure 3C). However, the number of bone marrow cells and LSK cells in the DP and WP supplemented mice was no longer significantly different from that of the Con group on 28 days after BMT, the NP group was still significantly lower than the Con group (*p* < 0.05; Figure 3A,C). To understand the status of hematopoietic stem/progenitor cells differentiation, we then tested varying populations of stem/progenitor cells from the LSK compartments. We did not find any significant difference in the primitive long-term hematopoietic stem cells between different treatment groups (Figure 3D). However, there were higher numbers of short-term hematopoietic stem cells in the DP group compared with the NP and WP groups (*p* < 0.01, *p* < 0.05; Figure 3E). We noticed a similar trend in the primitive multipotent progenitors among the DP and NP groups (*p* < 0.05; Figure 3F).

### 3.4. Thymus Re-Establishment Post-BMT

**Figure 4:**
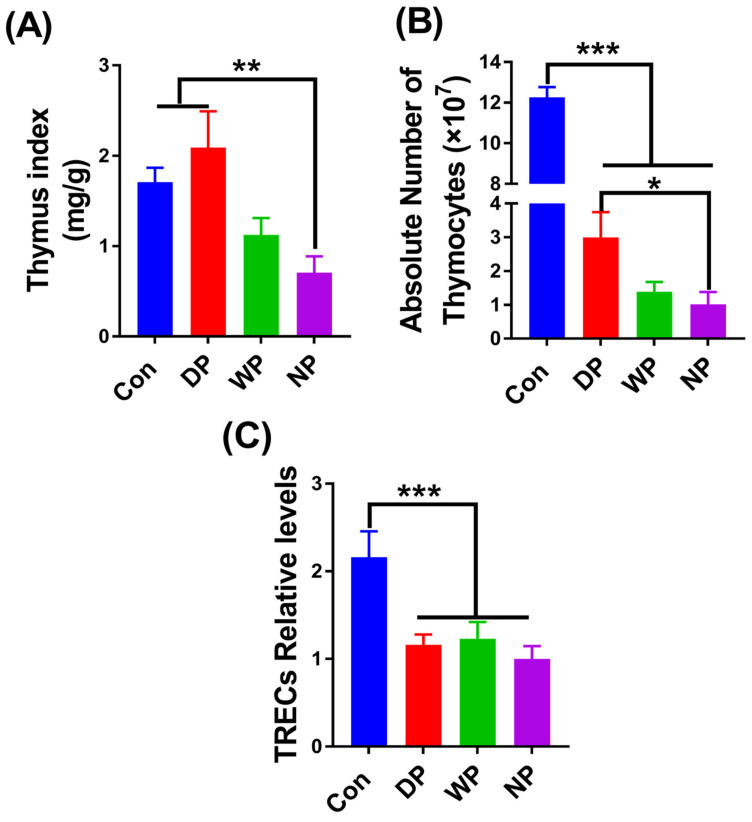
Thymus index, thymocyte count, and thymic recent output function were analyzed on day 28 post-BMT. Mice were euthanized before removal of the thymus for measuring thymus index (**A**); a single-cell suspension was obtained to measure the number of thymocytes (**B**); DNA was extracted from whole blood using a genomic DNA extraction kit (TIANMO, China). A relative quantitative method was used to evaluate the expression level of TRECs on day 28 after BMT (**C**). Data are presented as mean ± SEM (*n* = 5–7). Results are representative of at least two independent experiments. *** *p* < 0.001, ** *p* < 0.01, * *p* < 0.05.

Thymic tissues from transplanted mice on day 28 were further collected and analyzed for thymic size, thymocyte count, and thymic output function, to test whether protein supplementation could protect thymic function and promote thymocyte [38]. Interestingly, the thymus index and the thymocyte count in the DP group were 3-fold higher than their NP counterparts (*p* < 0.01, *p* < 0.05, respectively; Figure 4A,B). A higher thymus index and thymocyte count in the WP group were also observed, but the difference did not reach statistical significance. Expression of TRECs, a biomarker for thymic recent output function [39], did not differ in peripheral blood samples between the transplantation groups but was significantly lower than in the Con group (Figure 4C). However, we observed relatively higher expression levels of TRECs in the DP and WP groups.

### 3.5. Splenic Subset Recovery Post-BMT

**Figure 5:**
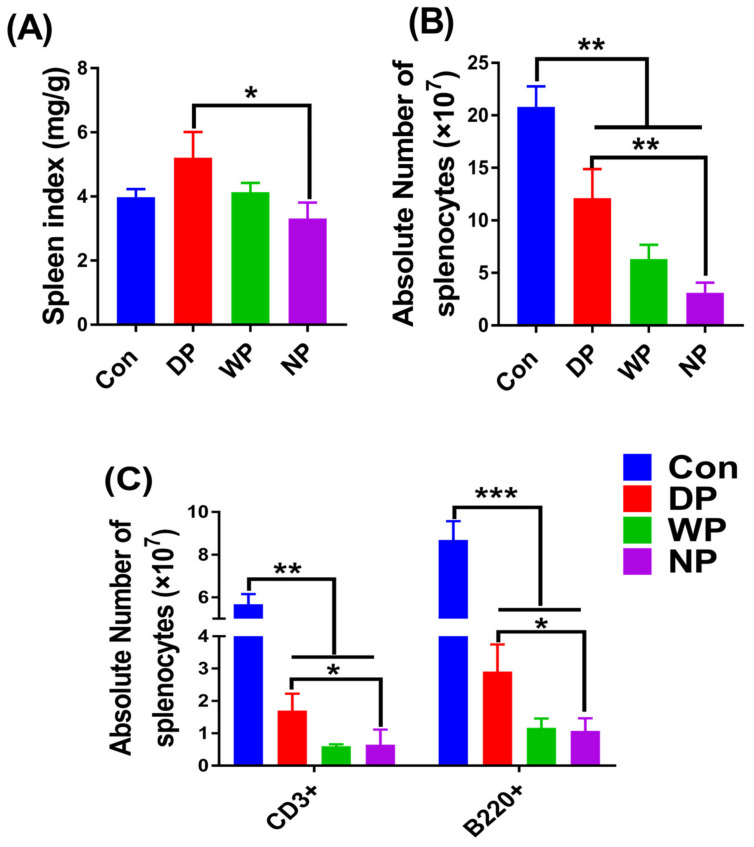
Spleen index, splenocytes, and splenic subsets count were analyzed on day 28 post-BMT. Mice were euthanized and the spleen was removed for the measurement of spleen index (**A**); a single-cell suspension was obtained to measure the number of splenocytes (**B**); splenic subsets were determined by well-defined cell surface markers (**C**). Data are presented as mean ± SEM (*n* = 5–7). Results are representative of at least two independent experiments. *** *p* < 0.001, ** *p* < 0.01, * *p* < 0.05.

The spleen, the largest immune organ and the central hub for cellular and humoral immunity, contains many lymphocytes. Myeloablative therapy, such as lethal irradiation, prior to BMT, leads to damage of the spleen [40]. Malnutrition impairs spleen function [41], which may delay immune reconstitution or to the repair of damage. Thus, spleen recovery is an essential part of immune reconstitution. Here, we analyzed the spleen index, the absolute number of splenocytes, and the population of splenic subsets, including T and B cells, on day 28 post-BMT to assess the effect of protein supplementation on spleen recovery (Figure S4). We found that protein intervention enhanced spleen weight restoration, and DP supplementation had the most pronounced effect (*p* < 0.05; Figure 5A). The absolute numbers of splenocytes in the DP and WP groups increased relative to the NP group, and the DP group exhibited the most obvious increase (*p* < 0.001; Figure 5B). To understand the characteristics of spleen recovery reflected in the increased populations of splenic T and B cells, we counted the absolute number of splenocytes on day 28 post-BMT. Similarly to the previous results of spleen index and splenocyte count, the numbers of T and B lymphoid cells in the DP supplemented group were higher than in the NP group (*p* < 0.05; Figure 5C). This finding implies that soy–whey protein supplementation had a surprisingly beneficial effect on immune recovery in the spleen after BMT.

### 3.6. Biochemical Indicators in Serum

**Figure 6:**
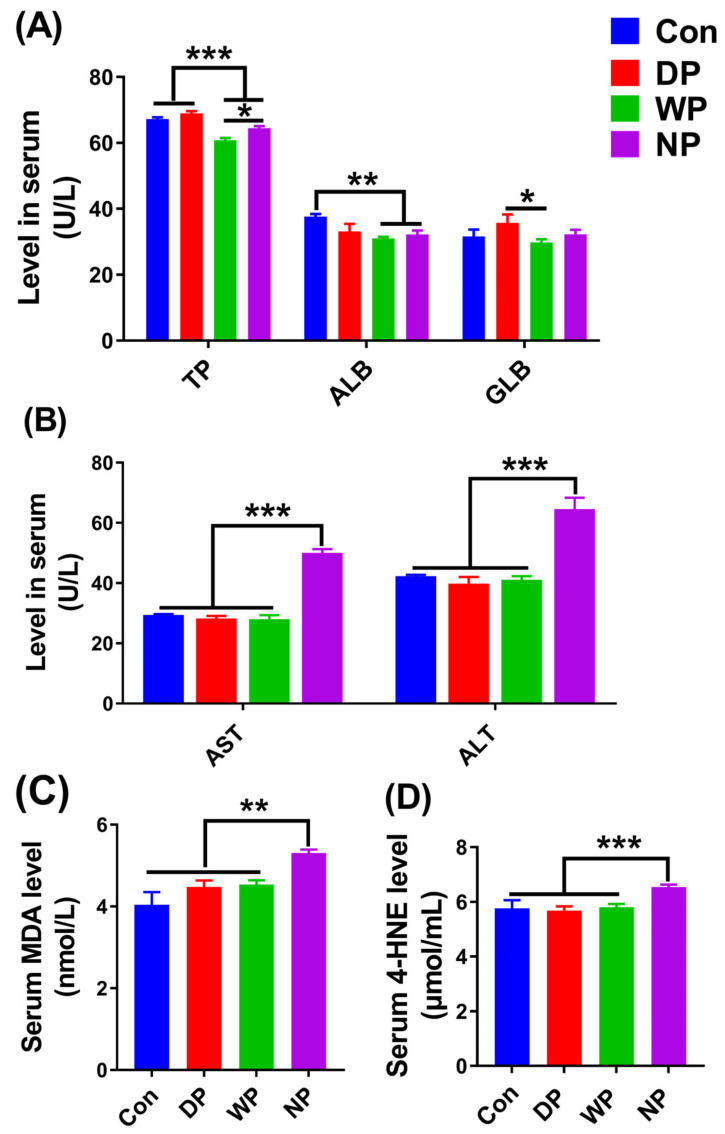
Biochemical indicators in serum on day 14 post-BMT. (**A**) Concentrations of albumin (ALB), globulin (GLB), and total protein (TP). (**B**) Concentrations of alanine aminotransferase (ALT) and aspartate aminotransferase (AST). (**C**) Concentrations of malondialdehyde (MDA). (**D**) 4-hydroxynonenal (4-HNE). Data are presented as mean ± SEM (*n* = 5–7). Results are representative of at least two independent experiments. *** *p* < 0.001, ** *p* < 0.01, * *p* < 0.05.

Serum protein content often serves as a crucial clinical prognostic biomarker to assess nutritional status after BMT and to monitor the effects of nutrition support [42]. We measured the levels of total serum protein, including albumin and globulin, on day 14 following BMT. The level of total serum protein in the DP group was improved compared with the WP and NP groups (*p* < 0.001; Figure 6A), and no significant difference was seen with the Con group, even though levels in the DP group where slightly higher. In addition, the concentrations of albumin and globulin in the DP group had varying increments compared with the other groups (Figure 6A). Serum protein content measurements indicated that DP supplementation exhibited the best effect in alleviating the malnutrition of mice following BMT.

Per-conditioning, such as lethal irradiation treatment prior to BMT, can result in cell and tissue injury from oxidative stress [43,44], which may lead to delayed immune reconstitution and poor clinical outcomes. Body oxidative stress indicators MDA and 4-HNE, and liver injury indicators ALT and AST, can be used to detect cell and tissue injury. Thus, we gathered serum samples from day 14 post-BMT mice to measure the concentrations of those indicators. Interestingly, we found lower concentrations of ALT, AST, MDA, and 4-HNE in sera from the DP and WP groups compared with the NP group (*p* < 0.01; Figure 6B–D). The contents of ALT, ASL, MDA and 4-HNE in the DP and WP groups were similar to those in the Con group, and there was no significant difference. These findings suggested that DP and WP supplementation following BMT may protect the liver from radiation-induced injury and improve antioxidant enzyme activity.

## 4. Discussion

Malnutrition is a common clinical complication of cancer patients, especially leukemia patients who undergo radiotherapy, chemotherapy, and BMT. Importantly, poor nutritional status likely increases the risk of early death and lengthens the hospitalization period [45]. In addition, poor nutrition delays hematopoiesis and immune reconstitution and increases susceptibility to high-risk opportunistic infections [46] and sudden relapse [47]. Whether a patient’s preoperative and intraoperative nutritional status affects immune reconstruction after transplantation is a serious concern for clinicians, patients, and their caregivers [48]. The post-BMT metabolic disorder primarily affects protein metabolism, energy balance, and micronutrient absorption [49]. A negative nitrogen balance, caused by an imbalance between protein synthesis and degradation, is also usually observed in BMT patients [50,51]. Well-balanced nutrition is indispensable for immune and bone marrow reconstruction [21]. However, few investigators have focused on the effects of soy–whey blended nutrition support in hematopoiesis and immune reconstitution.

We used an animal model to measure the effects of a soy–whey protein blend on hematopoietic and immune reconstitution after BMT. We found that soy–whey blended and single whey protein improved the weight recovery of mice post-BMT (Figure 1). We made similar conclusions for all types of protein supplements; however, DP supplementation exhibited the most significant positive effects (Figure 2 and Figure 3). These phenomena could be explained by the rapid expansion of c-kit^+^ progenitor cells, short-term hematopoietic stem/progenitor cells, and multipotent progenitors in DP-treated mice (Figure 3). These data suggested that post-BMT, DP supplementation promotes hematopoietic stem/progenitor cell differentiation by reverting overall malnutrition. Protein malnutrition may also result from either the inadequate consumption of branched-chain amino acids (BCAAs) or a BCAA metabolism imbalance [52,53]. Wilkinson et al. reported that reduced BCAA levels inhibited hematopoietic stem cell proliferation and survival in vitro and rendered these cells incapable of sustaining normal physiological activity in vivo [54]. Likewise, dietary BCAA depletion could lead to a significant decrease in WBC and RBC counts in normal mice [55]. Because soy and whey protein extracts contain abundant BCAAs [56,57], post-BMT DP supplementation was likely to reverse BCAA-deficits and promote survival and the proliferation of HSC and WBC populations [58,59]. In addition, protein malnutrition may lead to aberrant structural and cellular changes in the hematopoietic microenvironment, thereby inducing bone marrow atrophy, loss of HSC population, and an impaired immune response [60]. In agreement with these ideas, we found an increased serum protein content and HS/PC count in DP-supplemented mice (Figure 6A), which indicated that DP intervention reversed protein malnutrition and restored the hematopoietic microenvironment and HSC proliferation and differentiation. Belkaid and Hand [61] highlighted key effects of gut microbiota on mucosal and systemic immunity. The gut microbiota not only facilitates the generation of HS/PCs [62,63] but also, in an animal model, the microbiota facilitated hematopoietic recovery following BMT by improving dietary energy intake [64]. Moreover, intestinal flora depletion resulted in the impairment of hematopoiesis and lymphoid and myeloid differentiation [64]. Myeloablative radiation treatment prior to BMT with the background effects of malnutrition has been linked to abnormal changes in intestinal flora [65,66]. Furthermore, both soy and whey protein increased the diversity of intestinal flora [67,68] and relieved malnutrition, which might explain why we observed enhanced hematopoietic reconstitution in DP-supplemented post-BMT mice. However, further in-depth investigations are necessary to support these possibilities.

We observed faster recovery of the spleen and thymus that might have been a consequence of DP-supplementation post-BMT (Figure 4 and Figure 5). The thymus is the core place for T cell proliferation and differentiation. Xu et al. reported that irradiation-induced thymic damage was the primary reason for prolonged T cell reconstitution [11]. However, a good thymic architecture and microenvironment are critically important for efficient immune recovery. Thymic atrophy and thymocyte count decreased after BMT in our study. Interestingly, the thymus index and the absolute number of thymocytes significantly increased in the DP group. These results were likely attributable to the improvement of nutrition by DP because post-BMT mice might experience protein malnutrition. Ortiz et al. showed that malnourished rats had higher thymocyte apoptosis, a lower thymus weight, and lower thymocyte count compared with well-nourished animals [41]. The thymus is the lymphoid tissue most sensitive to protein-energy malnutrition, and the extent of atrophy represents the severity of malnutrition [69]. Thus, we observed that the nutritional condition of post-BMT mice was improved by DP supplementation because DP supplementation prevented a thymocyte decrease and thymus atrophy and promoted a fast recovery of the thymus.

Both irradiation and malnutrition can injure the spleen, which is manifested by a shrunken spleen organ in post-BMT mice [41,70]. Supplementation with DP increased the number of splenocytes, including splenic subsets of T and B cells, and finally enlarged the size of the organ. These increases suggested that DP supplementation hastened the repair of spleen injury and improved immune reconstitution by alleviating malnutrition and/or decreasing oxidative stress. Ortiz et al. reported that malnutrition led to reductions in spleen weight and splenocyte count [41]; in our study, these reductions were likely recovered by improving nutrition with DP supplementation. In addition, chemotherapy and/or radiotherapy prior to BMT can be associated with increased oxidative stress, the generation of cytotoxic free radicals, and depletion of antioxidants, all of which may induce fatal organ injury and exert negative effects on immune reconstitution [71,72]. Whey protein has a relatively high amount of cysteine. Cysteine determines the synthesis rate of the antioxidant glutathione [30,73,74]. Tosukhowong et al. showed that whey protein supplementation improved the ratio of reduced to oxidized glutathione in plasma of patients with Parkinson’s disease [30]. Moreover, Aoki et al. found that soy protein extract exhibited antioxidant activity against paraquat-induced oxidative stress in a rat model of Parkinson’s disease [29]. Therefore, in our study, either post-BMT DP or WP supplementation could reduce the extent of damage to the spleen and other organs caused by radiation-induced oxidative stress. This conclusion was supported by the low concentrations of MDA and 4-HNE in the mouse sera. As mentioned earlier, post-BMT, DP or WP supplementation either improved nutrition (Figure 6A) or enhanced antioxidant capacity (Figure 6B,C), which was beneficial for the repair of spleen injury and acceleration of immune reconstruction. However, the possible beneficial effects of protein therapy on spleen and thymus recovery following BMT require additional investigation.

We found a significantly increased serum protein content in DP-supplemented mice and reduced concentrations of MDA, 4-HNE, ALT, and AST in DP- and WP-supplemented mice (Figure 6). From a clinical perspective, Albumin concentration is a factor determining the length of hospitalization, infection susceptibility, and in-patient death [75,76]. Although we did not find any obvious differences in serum albumin concentration between the groups, higher concentrations of total protein, albumin, and globulin were observed in the DP-supplemented group, which was in agreement with previous findings and suggest that DP supplementation boosts the concentrations of serum protein components to various degrees [77]. Importantly, major biomarkers of liver injury, such as AST and ALT, had lower concentrations in DP- and WP-supplemented mice. MDA and 4-HNE are the end products of oxidative stress-induced lipid peroxidation [78,79]; lower MDA and 4-HNE concentrations demonstrated diminished oxidative stress and lipid peroxidation following protein therapy.

Despite the evidence that soy–whey protein nutritional supplementation had a favorable effect on hematopoiesis and immune reconstitution in allogeneic transplanted mice, there were still several limitations in our study. First, the immune and hematopoietic recovery status of the experimental mice was not monitored for a more extended periods of time, such as two months or even a longer experimental design. Second, the soy–whey blended protein mixture was a complex food and nutrients system full of nutritional compounds, and we did not define the composition and precise active principle responsible for the beneficial activities of the DP in BM allogenic transplant mice. We detected the protein contents, amino acids profiles, and even proteomics of whey and soy protein before the study, and clarified that soy and whey proteins are rich in branched-chain amino acids. We also found that the two sources of proteins are complementary in amino acids profiles and identified 1713 proteins and 728 proteins from soy and whey proteomes, respectively. However, determining the exact functional composition will require designing more complex and systematic experiments. The third molecular mechanisms of soy–whey double protein with soybean and milk providing better effects on hematopoiesis and immune reconstitution than single whey protein, still requires further research to fully understand. Severe protein-energy malnutrition (PEM) and skeletal muscle wasting are also commonly observed in patients with acute leukemia. The faster the hematopoietic recovery and the immune reconstitution, the lower the risk of recurrence, infection, and even death for patients post-BMT. Therefore, on the basis of this study, we provided insights on the suggested patient diets and precautions during the recovery period post BMT. First of all, the importance of protein nutrition support should be made clear whether in the hospital or once a patient returns home from the hospital. This is because some patients usually ignore the significance of protein nutritional supplementation after returning home, especially those with a poor family financial status in many regions. Secondly, under the condition of reasonable protein nutrient intake, we suggested that it is advisable to select a blended protein supplement containing a high proportion of soy protein and whey protein for patients after BMT.

In conclusion, we provided crucial evidence that soy–whey blended DP supplementation following BMT in mice improved body nutrition, the engraftment success rate, rate of hematopoiesis recovery and immune system reconstitution. Soy–whey supplementation also increased the sub-populations of HS/PCs, such as c-kit+, ST-HSCs, and MPPs. We also observed that DP supplementation led to the best restoration of the thymus and spleen. Thus, DP may be used in clinic as an effective nutritional supplement for BMT patients.

## Data Availability

The data presented in this study are available on request from the corresponding author.

## Supplementary Materials

The following supporting information can be downloaded at: https://www.mdpi.com/article/10.3390/nu14153014/s1, Figure S1: Donor chimerism; Figure S2: Analysis of Peripheral Blood Lymphocyte subsets by Flow Cytometry; Figure S3: Flow Cytometry analysis of HSCs after BMT; Figure S4: Flow Cytometry analysis of Splenic lymphoid subsets.

## Abbreviations

BMT	Bone marrow transplantation
WP	whey protein
WBC	white blood cells
GVHD	graft-versus-host disease
LSK	Lin-Sca1^+^c-kit^+^
ST-HSCs	short-term HSC cells
LMPs	lymphoid myeloid progenitors
EAAs	essential amino acids
DP	soy–whey blended double protein
TBI	total body irradiation
RBC	red blood cell
HS/PCs	hematopoietic stem/progenitor cells
LT-HSCs	long-term HSC cells
MPPs	primitive multipotent progenitors
TRECs	T cell receptor excision circles
BCAAs	branched-chain amino acids
