# Supplementary material for: Effects of Soy–Whey Protein Nutritional Supplementation on Hematopoiesis and Immune Reconstitution in an Allogeneic Transplanted Mice
Source: Nutrients. 2022 Jul 22;14(15):3014. doi: 10.3390/nu14153014 (PMC9332233; doi:10.3390/nu14153014)
Supplement: Supplementary file 1 [file nutrients-14-03014-s001.zip › nutrients-1819468-supplementary.pdf]

Supplementary Material

Effects of Soy–Whey Protein Nutritional Supplementation on Hematopoiesis and Immune Reconstitution in an Allogeneic Transplanted Mice

Xiaoliang Wu, Qinghua Hou, Zhenyu Zhao, Jing Wang, Yanzhi Guo, Lingang Lu * and Juan Han *

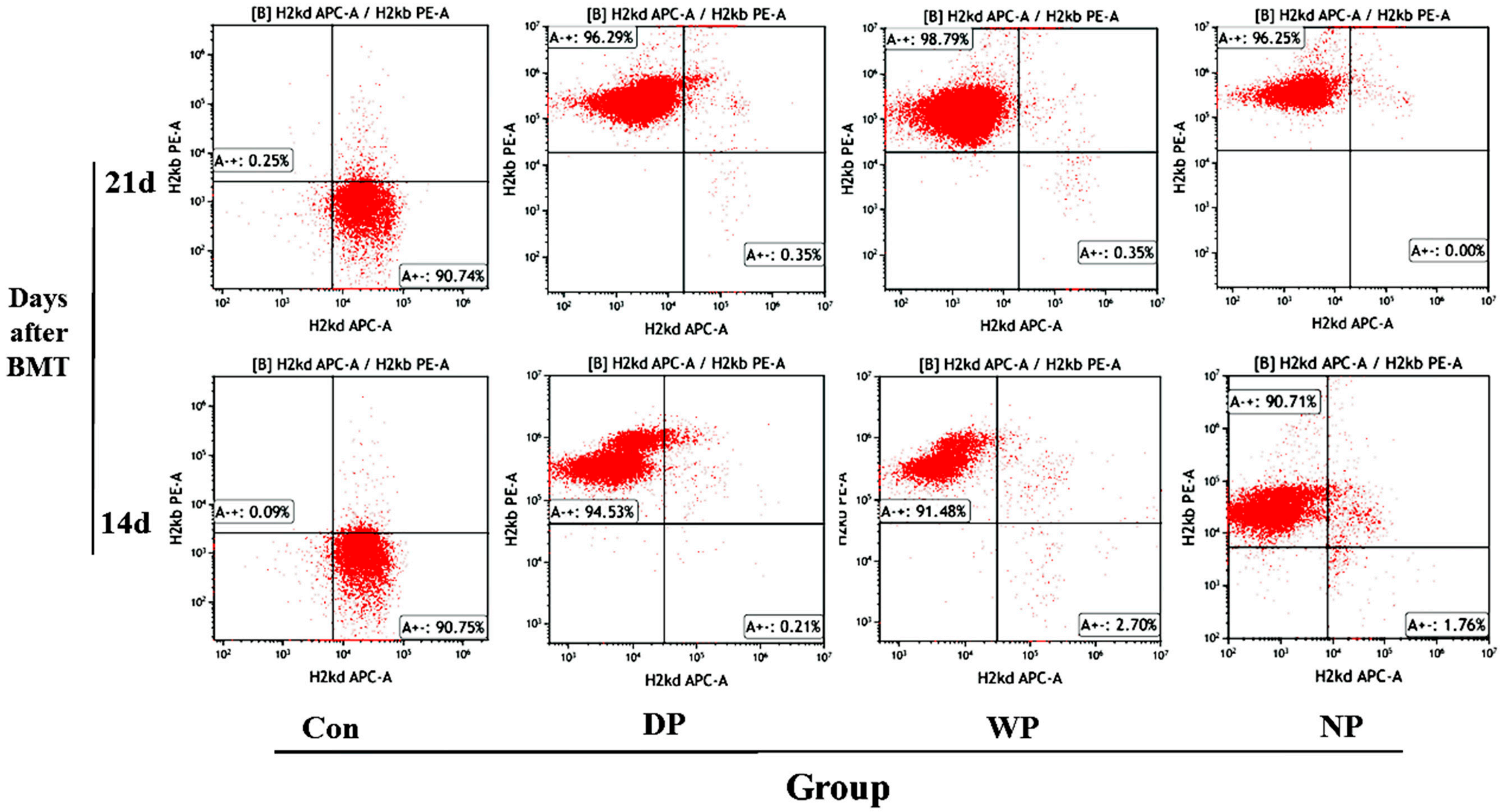

Supplementary Figure S1. Donor chimerism was measured using anti-mouse H-2Kd (FITC) and H-2Kb (PE) on days 14 and 21 (n=6).

A

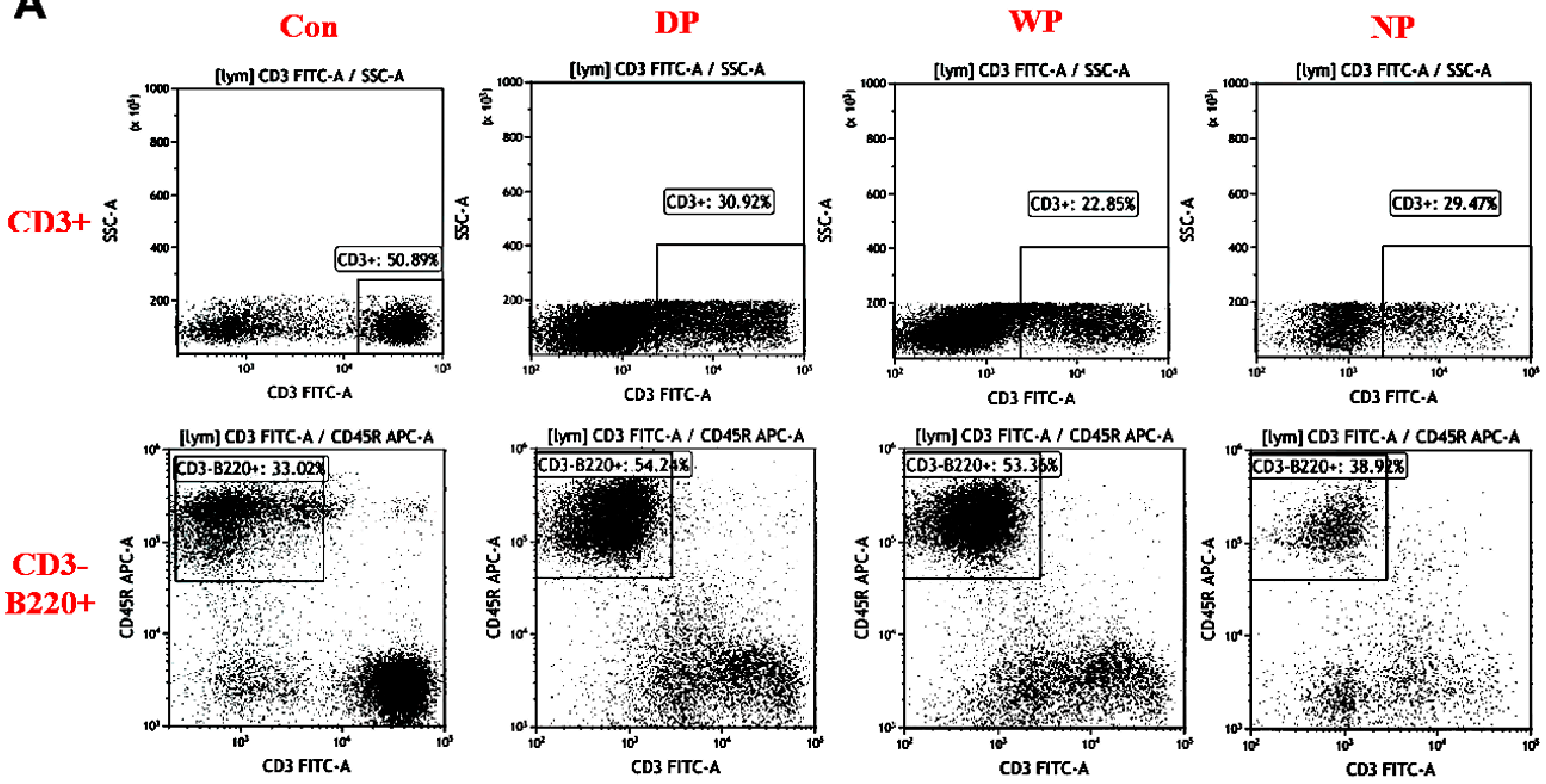

Con
DP
WP
NP
CD3+
CD3-
B220+
[lym] CD3 FITC-A / SSC-A
SSC-A
(x 10³)
CD3 FITC-A
CD3+: 50.89%
CD3+: 30.92%
CD3+: 22.85%
CD3+: 29.47%
[lym] CD3 FITC-A / CD45R APC-A
CD45R APC-A
CD3-B220+: 33.02%
CD3-B220+: 54.24%
CD3-B220+: 53.36%
CD3-B220+: 38.92%

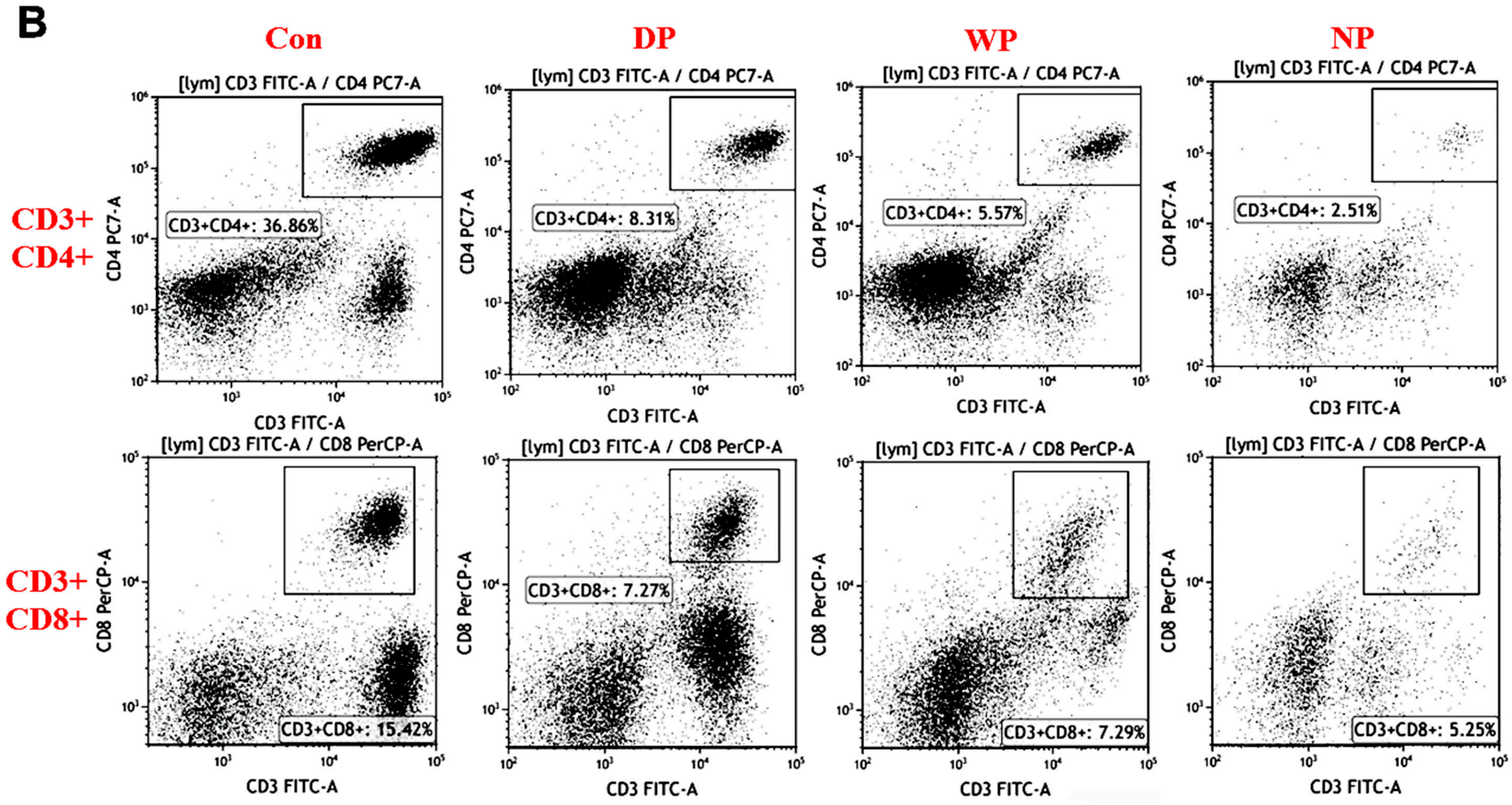
B
Con
DP
WP
NP
CD3+
CD4+
CD3+
CD8+
[lym] CD3 FITC-A / CD4 PC7-A
CD3+CD4+: 36.86%
CD3+CD4+: 8.31%
CD3+CD4+: 5.57%
CD3+CD4+: 2.51%
CD4 PC7-A
CD3 FITC-A
[lym] CD3 FITC-A / CD8 PerCP-A
CD3+CD8+: 15.42%
CD3+CD8+: 7.27%
CD3+CD8+: 7.29%
CD3+CD8+: 5.25%
CD8 PerCP-A

C

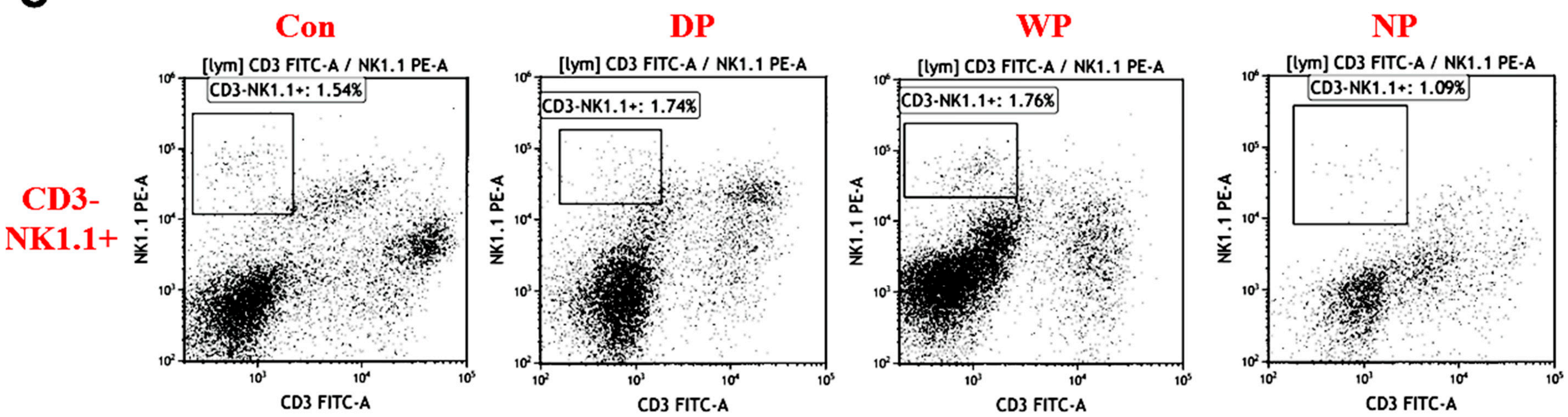
Con
DP
WP
NP
CD3-NK1.1+
[lym] CD3 FITC-A / NK1.1 PE-A
CD3-NK1.1+: 1.54%
CD3-NK1.1+: 1.74%
CD3-NK1.1+: 1.76%
CD3-NK1.1+: 1.09%
NK1.1 PE-A
CD3 FITC-A

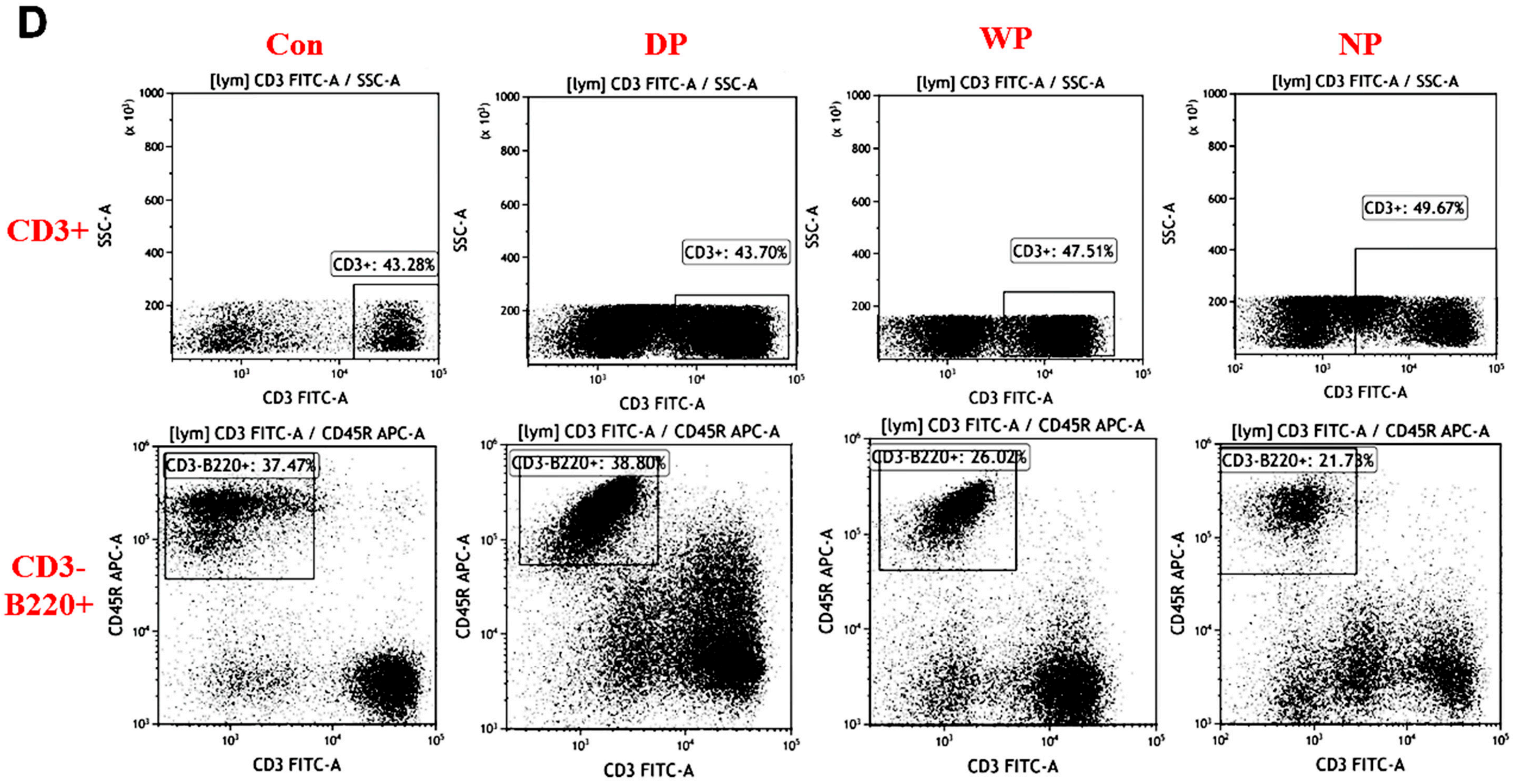
D
Con
DP
WP
NP
CD3+
CD3-
B220+
[lym] CD3 FITC-A / SSC-A
CD3+: 43.28%
CD3+: 43.70%
CD3+: 47.51%
CD3+: 49.67%
SSC-A
CD3 FITC-A
[lym] CD3 FITC-A / CD45R APC-A
CD3-B220+: 37.47%
CD3-B220+: 38.80%
CD3-B220+: 26.02%
CD3-B220+: 21.73%
CD45R APC-A

E

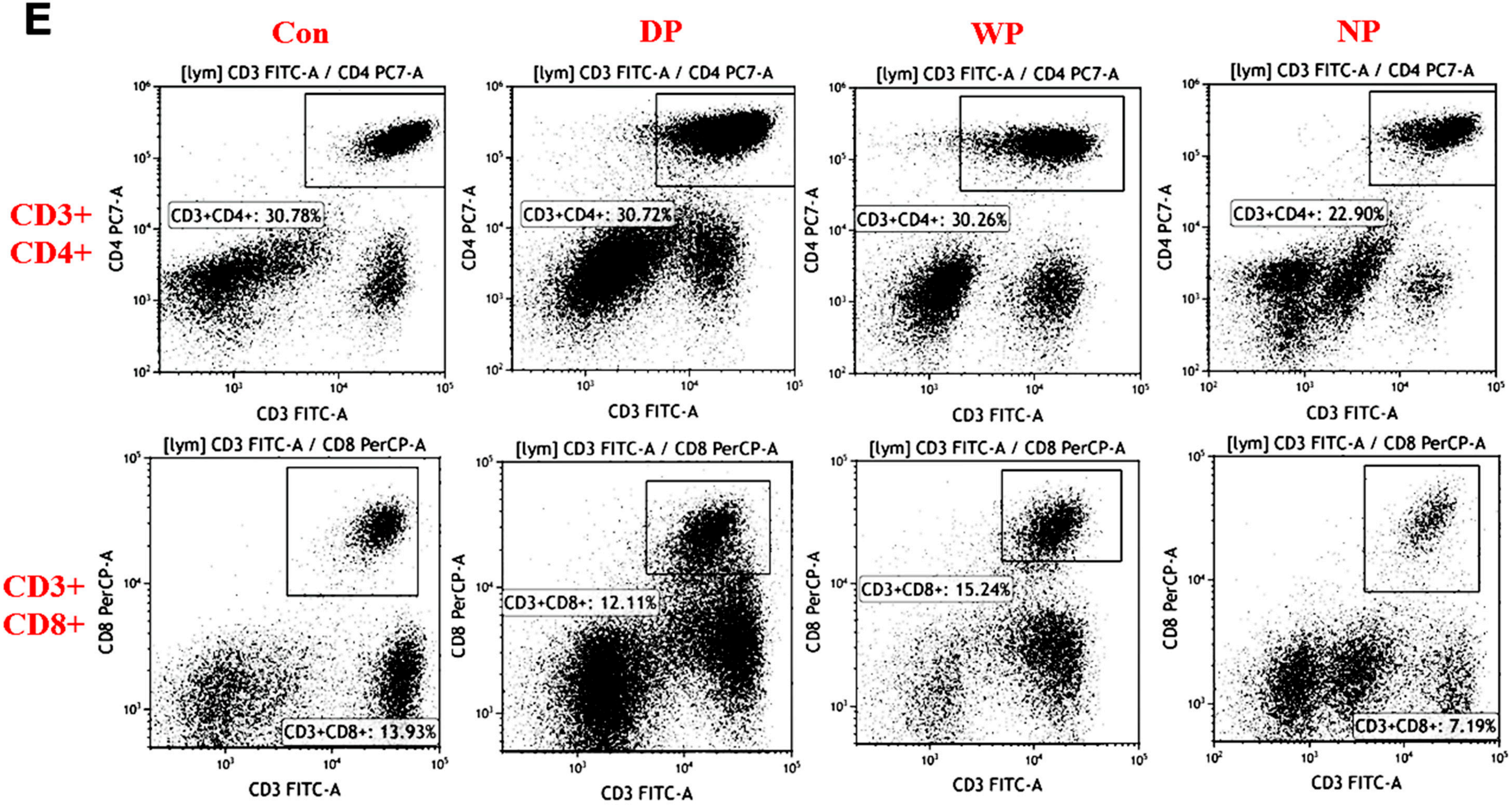

Con
DP
WP
NP
CD3+ CD4+
CD3+ CD8+
[lym] CD3 FITC-A / CD4 PC7-A
CD3+CD4+: 30.78%
CD3+CD4+: 30.72%
CD3+CD4+: 30.26%
CD3+CD4+: 22.90%
CD4 PC7-A
CD3 FITC-A
[lym] CD3 FITC-A / CD8 PerCP-A
CD3+CD8+: 13.93%
CD3+CD8+: 12.11%
CD3+CD8+: 15.24%
CD3+CD8+: 7.19%
CD8 PerCP-A
CD3 FITC-A

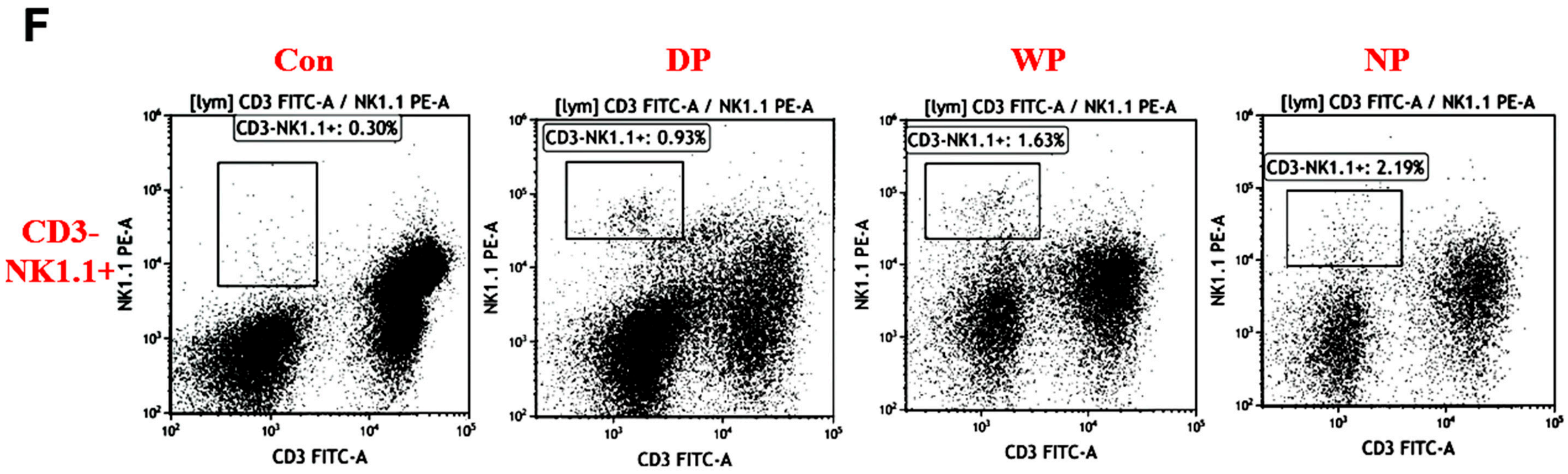

Supplementary Figure S2. Analysis of Peripheral Blood Lymphocyte subsets by Flow Cytometry on day 21(A-C), and 28 after BMT (D-F).

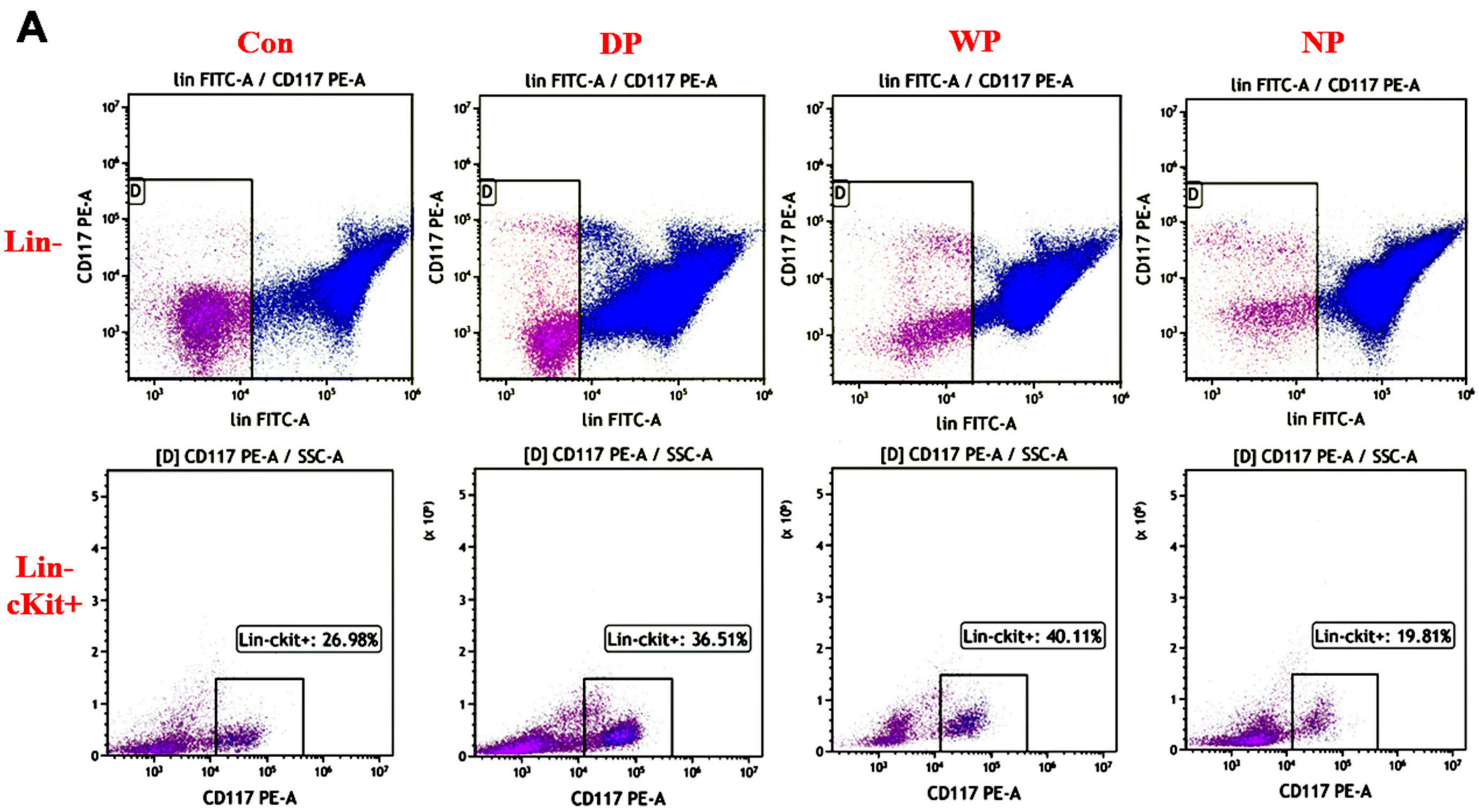
A
Con
DP
WP
NP
Lin-
Lin-
cKit+
lin FITC-A / CD117 PE-A
CD117 PE-A
lin FITC-A
[D] CD117 PE-A / SSC-A
CD117 PE-A
(x 10⁶)
Lin-ckit+: 26.98%
Lin-ckit+: 36.51%
Lin-ckit+: 40.11%
Lin-ckit+: 19.81%

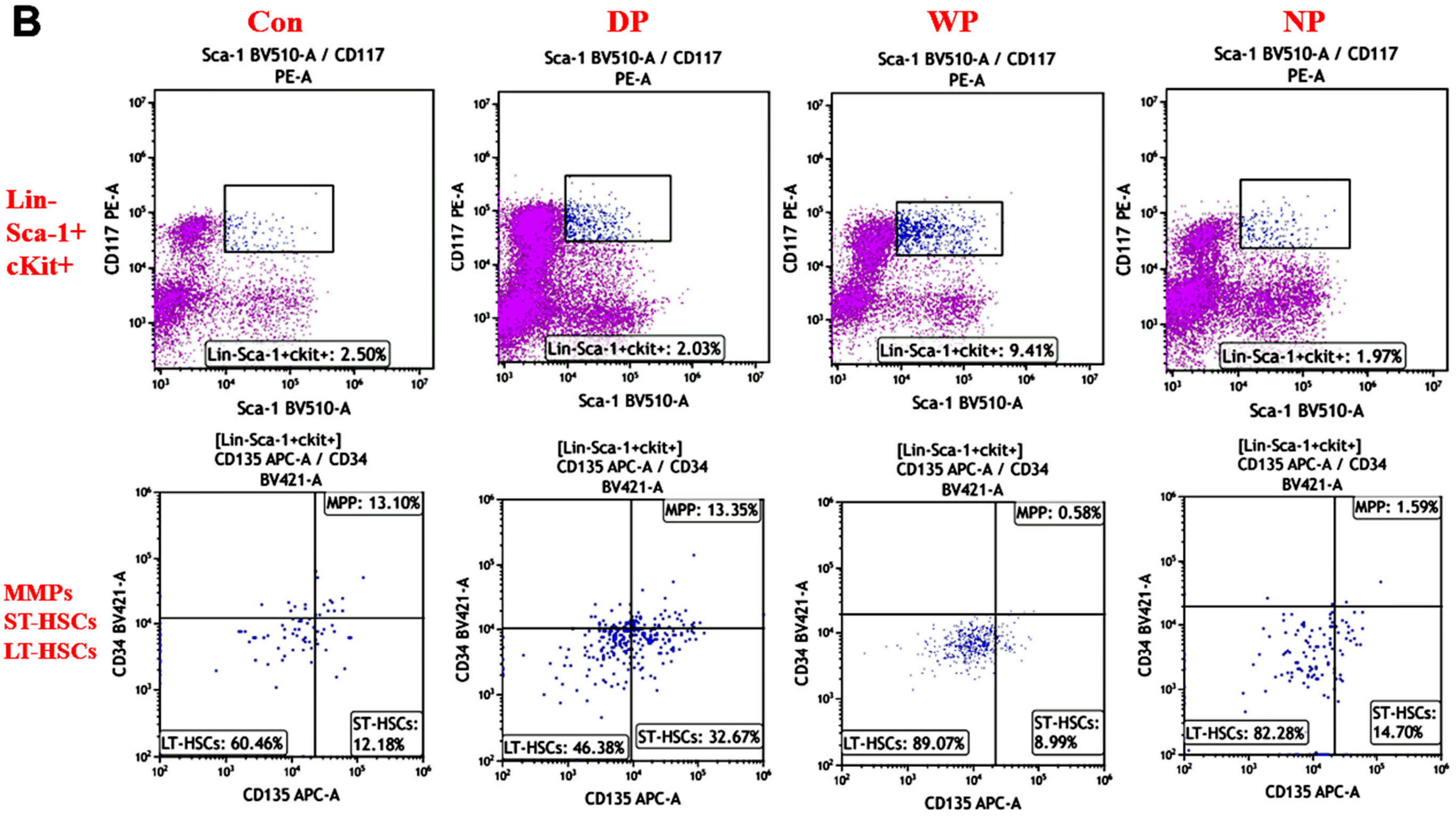

Supplementary Figure S3. Flow Cytometry analysis of c-kit cells (Lin-c-kit^{+}) (A); LSK, LT-HSCs (Lin-Sca1^{+}c-kit^{+}CD34^{-}Flk3^{-}), ST-HSCs (Lin-Sca1^{+}c-kit^{+}CD34^{+}Flk3^{-}), MPPs (Lin-c-kit^{+}Sca1^{+}CD34^{+}Flk3^{+}) populations(B) on day 28 after BMT.

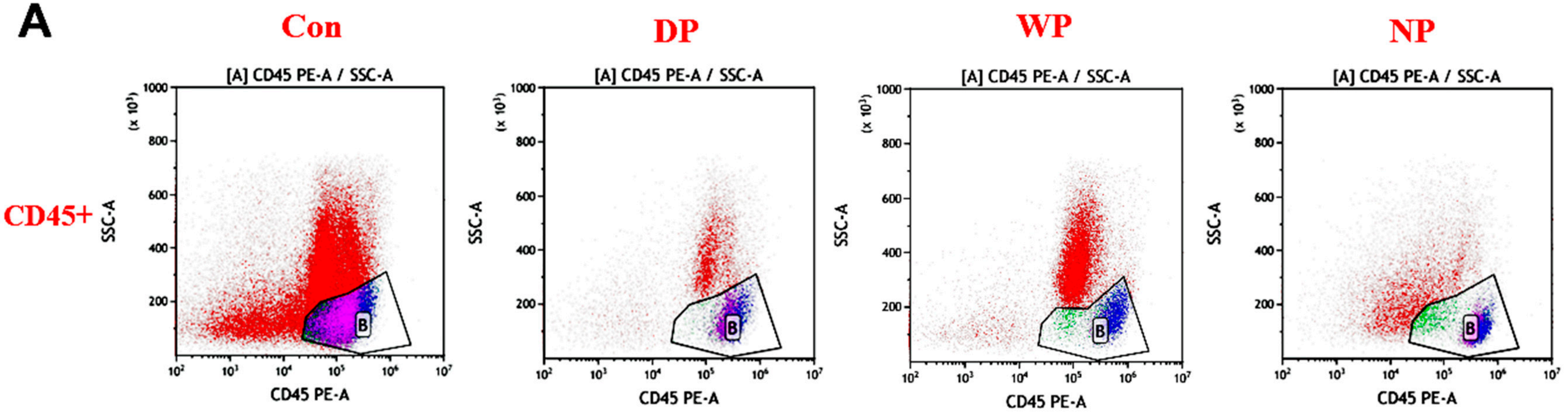
A
Con
DP
WP
NP
CD45+
[A] CD45 PE-A / SSC-A
SSC-A
CD45 PE-A
B

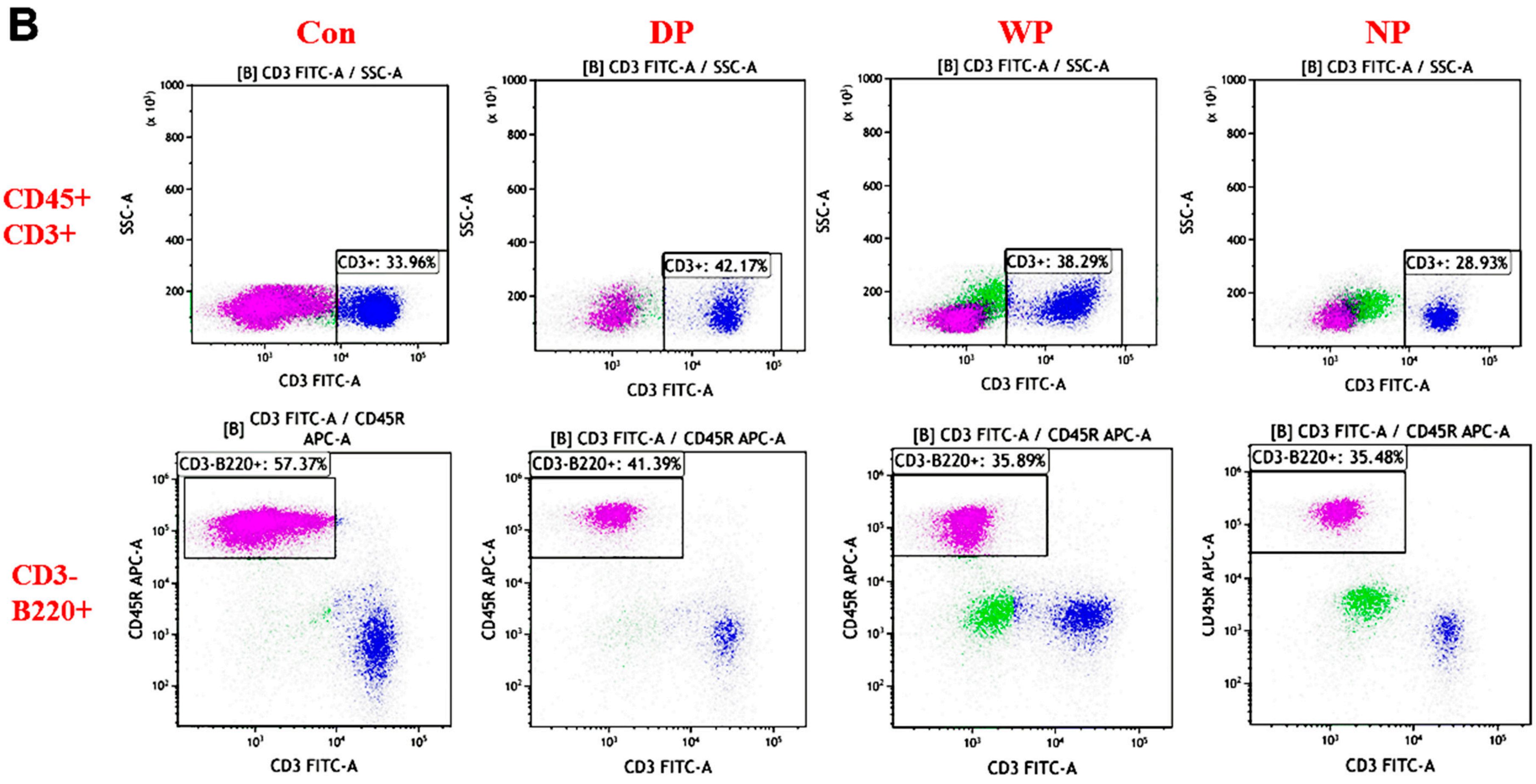

Supplementary Figure S4. Flow Cytometry analysis of Splenic lymphoid subsets on day 28 after BMT (A-B).
